# Correlation Patterns Among B7 Family Ligands and Tryptophan Degrading Enzymes in Hepatocellular Carcinoma

**DOI:** 10.3389/fonc.2020.01632

**Published:** 2020-09-03

**Authors:** Raghavan Chinnadurai, Rafaela Scandolara, Olatunji B. Alese, Dalia Arafat, Deepak Ravindranathan, Alton B. Farris, Bassel F. El-Rayes, Greg Gibson

**Affiliations:** ^1^Department of Biomedical Sciences, Mercer University School of Medicine, Savannah, GA, United States; ^2^Department of Hematology and Oncology, Winship Cancer Institute, Emory University, Atlanta, GA, United States; ^3^School of Biology, Georgia Institute of Technology, Atlanta, GA, United States; ^4^Department of Pathology and Laboratory Medicine, Emory University, Atlanta, GA, United States

**Keywords:** B7 family molecules, indoleamine 2,3 dioxygenase, hepatocellular carcinoma, T cell immunity, co-stimulation, co-inhibition, PD-L1

## Abstract

Mechanisms of dysfunctional T cell immunity in Hepatocellular Carcinoma (HCC) need to be well defined. B7 family molecules provide both co-stimulatory and co-inhibitory signals to T cells while tryptophan degrading enzymes like Indoleamine 2,3 dioxygenase (IDO) and Tryptophan 2,3 Dioxygenase (TDO) mediate tumor immune tolerance. It is necessary to identify their *in situ* correlative expression, which informs targets for combined immunotherapy approaches. We investigated B7 family molecules, IDO, TDO and immune responsive effectors in the tumor tissues of patients with HCC (*n* = 28) using a pathway-focused quantitative nanoscale chip real-time PCR. Four best correlative expressions, namely (1) B7-1 & PD-L2, (2) B7-H2 & B7-H3, (3) B7-2 & PD-L1, (4) PD-L1 & PD-L2, were identified among B7 family ligands, albeit they express at different levels. Although TDO expression is higher than IDO, PD-L1 correlates only with IDO but not TDO. Immune effector (Granzyme B) and suppressive (PD-1 and TGF-β) genes correlate with IDO and B7-1, B7-H5, PD-L2. Identification of the *in situ* correlation of PD-L1, PD-L2 and IDO suggest their cumulative immuno suppressive role in HCC. The distinct correlations among B7-1, B7-2, B7-H2, and B7-H3, correlation of PD-1 with non-cognate ligands such as B7-1 and B7-H5, and correlation of tumor lytic enzyme Granzyme B with IDO and PD-L2 suggest that HCC microenvironment is complexly orchestrated with both stimulatory and inhibitory molecules which together neutralize and blunt anti-HCC immunity. Functional assays demonstrate that both PDL-1 and IDO synergistically inhibit T cell responses. Altogether, the present data suggest the usage of combined immune checkpoint blocking strategies targeting co-inhibitory B7 molecules and IDO for HCC management.

## Introduction

Hepatocellular carcinoma (HCC) is one of the leading causes of cancer-related mortalities (1). Defining the immunological mechanisms contributing to HCC pathogenesis in the intrahepatic tumor microenvironment is of translational interest (2). Co-stimulatory, co-inhibitory (B7 family) pathways and enzymes of tryptophan degradation predominantly attenuate anti-tumor T-cell immunity. FDA-approved inhibitors of PD-L1 (B7-H1) & PD-L2 (B7-DC)-PD-1 interaction to revive exhausted anti-tumor T-cell immunity have shown benefit to cancer patients (3, 4). In addition, clinical trials are ongoing to test the efficacy of inhibitors of tryptophan degrading enzymes for cancer treatment (NCT03695250) (5–7). Early phase clinical trials are ongoing to test the effect of immunotherapy agents targeting B7 family and tryptophan degradation pathways on HCC (8–10). Importantly, inhibitors of PD-L1 (B7-H1) & PD-L2 (B7-DC)-PD-1 pathways are promising for anti-HCC management (11, 12). Defining the complex interaction among B7 family ligands and enzymes of tryptophan degradation pathways in the HCC microenvironment will inform additional checkpoint immune blocking strategies.

B7 family ligands are the dominant family of molecules providing co-stimulation and co-inhibition to T-cells. Accumulated evidence demonstrates that there are many members present in the B7 family, namely, B7-1, B7-2, PD-L1 (B7-H1), PD-L2 (B7-DC), B7-H2, B7-H3, B7-H4, B7-H5, B7-H6 and B7-H7 (13, 14). Receptors of the majority of the B7 family ligands have been defined on T-cells while some ambiguity exists in identifying definitive receptors of new members B7-H3, B7-H4, B7-H5, and B7-H7 (Table 1). Nevertheless, dysfunctional and exhausted T-cells co-express receptors of B7-family ligands, which suggest that a complex co-receptor and ligand interaction occur in a tumor microenvironment and confer dysfunctional immune response (15–18). The interaction of PD-L1/PD-L2 with PD-1 on T cells plays an important role in modulating tumor immunity (19). B7-H2 has been characterized as a co-stimulatory ligand for Inducible Costimulator (ICOS) and skews T-cell differentiation toward Th2 responses (20, 21). In contrast, B7-H3 is a negative regulator by preferentially affecting Th1 responses (22, 23). B7-H4 has been shown to play a major role in the negative regulation of T cell immunity (24, 25). B7-H5 or V-domain Ig Suppressor of T cell Activation (VISTA) is identified as both co-stimulatory (26) and co-inhibitory molecule (27). B7-H6 is highly expressed on many cancer conditions and interacts with NK-cell receptor NKP30 to induce activation while its significance on tumor immunity is yet to be defined (28). B7-H7 is a co-inhibitory molecule and inhibits CD4+ and CD8+ T cell functions (29). The pattern of correlative expressions of B7 family molecules and their interaction with the immune effectors of HCC microenvironment is currently unknown.

**Table 1:** B7 Family ligand and receptors.

**Ligand**	**Receptor**	**T Cell Fate**
B7-1	CD28	+
B7-1	CTLA4	–
B7-2	CD28	+
B7-2	CTLA4	–
PD-L1 (B7-H1)	PD-1	–
PD-L2 (B7-DC)	PD-1	–
B7-H2	ICOS	+
B7-H3	?	–
B7-H4	?	–
B7-H5	CD28H	±
B7-H6	NKp30	+*
B7-H7	?	–

* *NK cell fate*.

Indoleamine 2,3-dioxygenase (IDO) and tryptophan 2,3-dioxygenase (TDO) deplete tryptophan by converting it into the immunosuppressive catabolite, kynurenine (30–32). Although inconsistent results emerge in clinical trials targeting IDO (33), it needs to be further clarified on how IDO and/or TDO operate with B7 family ligands in modulating T-cell responses in the liver of patients with HCC. Important questions are: How are B7 family ligands expressed relative to each other in controlling T cell fate? How do B7 family ligands correlate among themselves in the tumor microenvironment of HCC? Do B7 family ligands co-express with IDO and TDO in an intricate intrahepatic tumor microenvironment? How do B7 family ligands, IDO and TDO correlate with immune responsive effector molecules in HCC? Better understanding of the underlying *in situ* associations of IDO/TDO and B7 family members will inform the rationale of translational development of specific checkpoint inhibitors for primary liver cancer management.

## Methods

### Patient Characteristics

The Emory University Institutional Review Board (IRB) approved the study. All consecutive cases of patients with HCC who were older than 18 years of age and treated between 2013 and 2015 at Winship Cancer Institute/Emory University Hospital system were identified and enrolled. All the patients (*n* = 28) who were included in the study had active HCC (Table 2).

**Table 2:** HCC patient characteristics.

	**Gender**	**Age**	**Race - A, AA, C, O**	**Diagnosis**	**AJCC stage**	**T**	**Serum AFP at diagnosis (ng/mL)**	**Tumor Grade**	**% Tumor**	**% Leukocytes**
1	Male	65	O-Hispanic	HCC and Cholangio		pT2	93.1	G2: Moderately differentiated	50	10
2	Male	45	O-African	HCC		pT1	4.9	G2: Moderately differentiated	40	5
3	Female	71	Caucasian	HCC	IIIA	pT3a	1.6	G2: Moderately differentiated	80	3
4	Male	57	Caucasian	HCC	I	pT1	2.2	G1: Well-differentiated	70	2
5	Female	67	Caucasian	HCC	I	pT1	1.4	G1: Well-differentiated	80	2
6	Male	62	Caucasian	HCC		pT2	7	G2: Moderately differentiated	60	2
7	Male	69	Caucasian	HCC		pT2	15.2	G2: Moderately differentiated	30	N/A
8	Male	60	Caucasian	HCC	I	pT1	20.1	G2: Moderately differentiated	70	2
9	Male	60	AA	HCC	I	pT1	354	G2: Moderately differentiated	60	5
10	Female	59	AA	HCC	II	pT2	17.7	G2: Moderately differentiated	80	2
11	Male	66	Caucasian	HCC		pT2	4.7	G2: Moderately differentiated	70	10
12	Male	75	Caucasian	HCC	I	pT1	4.9	G2: Moderately differentiated	90	3
13	Female	61	AA	HCC		pT1	>2,000	G2: Moderately differentiated	90	0.5
14	Male	68	AA	HCC	II	pT2	21.7	G1: Well differentiated	30	2
15	Male	55	Caucasian	HCC		pT2	17.4	G2: Moderately differentiated	70	5
16	Female	35	AA	HCC		pT1	3,969	G3: poorly differentiated	60	2
17	Male	67	Caucasian	HCC	II	pT2	64.6	G1: Well differentiated	60	5
18	Male	65	Caucasian	HCC	I	pT1	2.9	G2: Moderately differentiated	40	2
19	Female	44	Caucasian	HCC		pT1	5.3	G1: Well differentiated	50	2
20	Male	47	Caucasian	HCC		pT2	4.6	G2: Moderately differentiated	30	1
21	Male	65	Caucasian	HCC		pT1	6.3	G2: Moderately differentiated	N/A	3
22	Male	71	AA	HCC	II	pT2	23	G2: Moderately differentiated	50	N/A
23	Male	53	Caucasian	HCC		pT2	18.4	Other: Well to moderately differentiated	60	5
24	Male	53	Caucasian	HCC		pT1	18.8	G3: poorly differentiated	50	3
25	Male	64	Caucasian	HCC	II	pT2	214.9	G2: Moderately differentiated	70	3
26	Male	51	Caucasian	HCC		pT2	1,750.6	Other: moderately to poor differentiated	60	5
27	Male	67	AA	HCC		pT1	20.4	G2: Moderately differentiated	80	1
28	Male	59	Caucasian	HCC		pT2	10.1	G2: Moderately differentiated	90	2

### RNA Extraction and cDNA Preparation

Paraffin embedded tissue blocks from HCC patients were subjected to hematoxylin and eosin (H&E) staining to ensure that the tissue slices subjected to RNA analysis have adequate (minimum 30–40%) tumor volume. % Tumor volume and % leukocytes in tissue sections are given in Table 2. High Pure FFPE (formalin-fixed, paraffin-embedded) RNA Micro Kit was used to isolate RNA from the dissected paraffin slices according to the manufacturer instructions (Roche GmbH, Germany). Hundred to five-hundred nanogram of isolated RNA was used for total cDNA preparation (QuantiTect Reverse Transcription Kit, Qiagen, USA).

### Fluidigm Nanoscale PCR

Quantitative RT-PCR was performed using Fluidigm 48 × 48 nanofluidic arrays. Briefly, cDNA samples were pre amplified with 14-cycle PCR reaction for each sample with the combination of 100 ng cDNA and pooled primers as described by TaqMan Pre-Amp Mastermix (Fluidigm BioMark™) manufacturer's protocols. Two thousand three hundred four parallel qRT-PCR reactions were performed for each primer pair on each sample on a 48 × 48 chip array. Human cDNA library was used as a positive control. Amplification was detected in Eva Green detection assay on a Biomark I machine based on standard Fluidigm protocols as described previously (34, 35). Two independent primer pairs were used for each target (Table 3). Cycle of Threshold (CT) values were normalized based on the endogenous GAPDH & beta actin controls. CT value of each target was subtracted with the CT value of endogenous GAPDH & beta actin (Control CT values) of the respective samples to get delta CT values. The delta CT values were subjected to a second standardization by adding all the delta CT values with a constant CT value, which was derived from the total average of control CT values from all the samples. In the case of Osteopontin or Glypican normalization, GAPDH & beta actin normalized CT values were further normalized with Osteopontin or Glypican-3. The normalized CT values are expressed as an inverse (CT^−1^) to better graphically represent the increase or decrease in expression as described previously (36). Inverse CT values were obtained by dividing 1 with the appropriate normalized CT value. High or low inverse CT values represent high or low expression of target genes, respectively.

**Table 3:** Primer sequences of the targets used in nanoscale chip PCR.

**Target**	**Primer Pair**	**Forward**	**Reverse**
B7-1	1	GCTGTCCTGTGGTCACAATG	TGCCAGTAGATGCGAGTTTG
	2	CACCTCTCCTGGTTGGAAAA	TAAGGTAATGGCCCAGGATG
B7-2	1	GTATTTTGGCAGGACCAGGA	CTTGTGCGGCCCATATACTT
	2	GTATTTTGGCAGGACCAGGA	CGGCCCATATACTTGGAATG
PD-L1	1	TATGGTGGTGCCGACTACAA	TGACTGGATCCACAACCAAA
	2	CGAAGTCATCTGGACAAGCA	CTCTTGGAATTGGTGGTGGT
PD-L2	1	GGAACTTACTTTGGCCAGCA	GATGCAGAAGGGGATGAAAA
	2	AAGTCCAAGTGAGGGACGAA	GGCGACCCCATAGATGATTA
B7-H2	1	GTCCTGGACTGCTCTTCCTG	CCATCGCTCTGACTTCCTTC
	2	GTCCTGGACTGCTCTTCCTG	TCGCTCTGACTTCCTTCTCC
B7-H3	1	GTGGGGCTGTCTGTCTGTCT	TGATCTTTCTCCAGCACACG
	2	GTCTCATTGCACTGCTGGTG	TGATCTTTCTCCAGCACACG
B7-H4	1	GTCGGAGCAGGATGAAATGT	TGAGTTGCACGTTTTTCAGC
	2	GCTGAAAAACGTGCAACTCA	TAGCATTCCCCTTGCCTTTA
B7-H5	1	GGCAACTTCTCCATCACCAT	CAGTAGAGGCCGCTATCCAG
	2	GCATCGTAGGAATCCTCTGC	CCTGCCTTTGCTTGTAGACC
B7-H6	1	CACACCCCTGAATGACAATG	TGTTGAGGGGTTGGGAATAA
	2	CTGAAGGCACAGGGAACAGT	TTGATCCAGCAACAATCTGC
B7-H7	1	GACCATTTGGAAAGCCAAGA	CGCATTCCCATTTTGAATCT
	2	TAGAAGCCAGGAGGAGCAGA	CCAGGAGGGACACAACATCT
IFNγ	1	TTCAGCTCTGCATCGTTTTG	ATGGGTCCTGGCAGTAACAG
	2	TCATCCAAGTGATGGCTGAA	CTTCGACCTCGAAACAGCAT
TNFα	1	AACCTCCTCTCTGCCATCAA	GGAAGACCCCTCCCAGATAG
	2	GAGAAGGGTGACCGACTCAG	CCAAAGTAGACCTGCCCAGA
TGFβ	1	GTACCTGAACCCGTGTTGCT	CACGTGCTGCTCCACTTTTA
	2	AGCTCCACGGAGAAGAACTG	GTCCTTGCGGAAGTCAATGT
IL-10	1	GAGAACAGCTGCACCCACTT	TCTCGGAGATCTCGAAGCAT
	2	TTACCTGGAGGAGGTGATGC	GCCTTGATGTCTGGGTCTTG
IL-2	1	TCACCAGGATGCTCACATTT	GCACTTCCTCCAGAGGTTTG
	2	CCCAGGGACTTAATCAGCAA	ATGGTTGCTGTCTCATCAGC
IDO	1	GCCCTTCAAGTGTTTCACCAA	CCAGCCAGACAAATATATGCGA
	2	CCTGAGGAGCTACCATCTGC	GCTTGCAGGAATCAGGATGT
TDO	1	CAAATCCTCTGGGAGTTGGA	GTGCATCCGAGAAACAACCT
	2	GACGGCTGTCATACAGAGCA	CCTGGAACCTAGGCTCTTCC
Perforin	1	ACTCACAGGCAGCCAACTTT	GGGTGCCGTAGTTGGAGATA
	2	CCCTCTGTGAAAATGCCCTA	GGCTTAGGAGTCACGTCCAG
Granzyme B	1	ACTGCAGCTGGAGAGAAAGG	CTGGGCCTTGTTGCTAGGTA
	2	GGAGGCCCTCTTGTGTGTAA	TGCCATTGTTTCGTCCATAG
PD-1	1	TGCAGCTTCTCCAACACATC	CATGCGGTACCAGTTTAGCA
	2	GTGCCTGTGTTCTCTGTGGA	TTCTCTCGCCACTGGAAATC
Osteopontin	1	CATCACCTGTGCCATACCAG	GCCACAGCATCTGGGTATTT
	2	TGAAACGAGTCAGCTGGATG	GCTCTCATCATTGGCTTTCC
Glypican-3	1	CCAACATGCTGCTCAAGAAA	TCCATGTTCAATCGTGCTGT
	2	CTGGATGAGGAAGGGTTTGA	AGCCTCCAATGCACTCATCT

### T Cell Proliferation and Cytokine Secretion

Peripheral Blood Mononuclear Cells (PBMCs) were isolated from heparin-anticoagulated whole blood using a Ficoll-Paque PLUS density gradient (GE Healthcare Biosciences, Sweden), and cryopreserved in medium containing 90% fetal calf serum (HyClone) and 10% dimethyl sulfoxide (Corning, USA). Prior to the experiment, cells were thawed and rested overnight in a 37°C CO_2_ incubator. For proliferation assays, Carboxyfluorescein succinimidyl ester (CFSE) (Biolegend USA) labeled PBMC were stimulated for 4 days with plate bound 1 μg/ml anti-CD3 and anti-CD28 (Biolegend, USA) antibodies and 10ug/ml of PD-L1 Ig or Control Ig protein (Biolegend, USA) with varying concentrations of Kynurenine (Tocris, USA). Four days later, cells were stained with CD3-APCCy7 antibody (Biolegend, USA) and acquired in BD FACSAria instrument. Results were analyzed in Flow Jo software. Supernatants were analyzed for IFNγ using the kit, Human IFN gamma ELISA Ready-SET-Go according to the manufacture's instruction.

### Statistics

Data were analyzed with GraphPad Prism 5.0 software for statistics. An unpaired two-sided *t*-test was used to determine significance between the means of two groups, and a one-way ANOVA with Tukey's multiple comparison tests was used to compare multiple groups simultaneously. *P*-value < 0.05 was considered statistically significant. Linear regression analysis was performed using normalized CT values to obtain *R*^2^ and *P*-values. *R*^2^ = Goodness of Fit; P = Significance of the slope deviation from Zero. Correlations with *R*^2^ values above 0.5 with the *P*-value of < 0.05 were considered as the best correlation. Correlation with *R*^2^ values between 0.3 and 0.5 with the *P*-value of < 0.05 were considered as moderate correlations/statistical trend toward a correlation. Correlations with *R*^2^ values below 0.3 were considered relatively less significant.

## Results

### Differential Expression of B7 Family Ligands and Enzymes of Tryptophan Degradation in Patients With HCC

**Figure 1:**
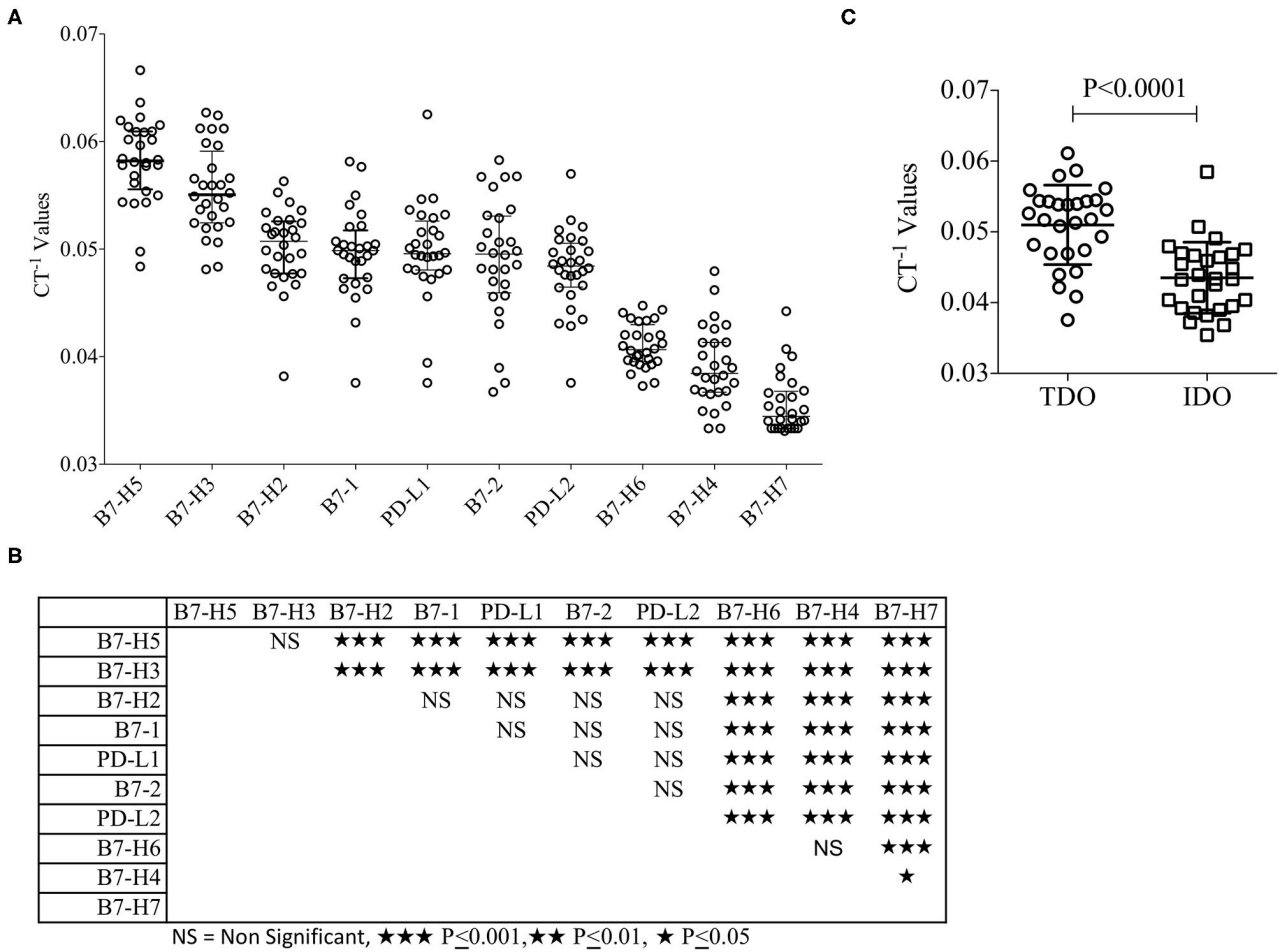
Relative expression of B7 familiy ligands and enzymes of Tryptophan degradation in HCC. Paraffin embedded liver tissue (10 micron) sections were collected in tubes and Hematoxylin and Eosin stained sections were reviewed to ensure tumor content. RNA was extracted and converted into cDNA which was used for the B7 Family ligand amplification in Fluidigm^TM^ nanoscale quantitative RT PCR. Each target is validated with two independent primer sets with the amplicon length of <100 base pairs. **(A)** Inverse CT values (CT^−1^) of B7 Family ligands derived from each patient were plotted with median and range. Median values were organized in hierarchical order of high to low expression. **(B)** One-way ANOVA and Tukey's multiple comparison test was performed to obtain *P*-values. Table shows the statistical significance of the median of CT^−1^ values of each B7 family ligand in comparison to others. **(C)** Inverse CT values (CT^−1^) of TDO and IDO derived from each patient were plotted with the median and range. CT values were normalized based on GAPDH & beta actin controls.

B7 family molecules are either co-stimulatory or co-inhibitory in stimulating and negating T-cell responses (Table 1). We have evaluated their relative expression in the hepatic tumor microenvironment in patients with HCC. Surgically resected liver tumor tissues of the patients with HCC (*n* = 28) were fixed with formalin and embedded in paraffin. Hematoxylin and Eosin (H&E) staining was performed for the inclusion criteria that the tissue slices subjected to RNA analysis have at least 30–40% tumor volume (Table 2). % Tumor volume and % leukocytes in tissue sections are given in Table 2. Patient #1 had a minute focus of cholangiocarcinoma on morphology which was excluded on histologic analysis. Total RNA was extracted from tissue sections, which was then converted into cDNA. cDNA samples were then used to perform Fluidigm nanoscale quantitative real-time chip PCR with two independent primer pairs for each target. Human cDNA library was used as a positive control to ensure the effectiveness of the primer pairs. The cycle of threshold (CT) values were normalized based on endogenous GAPDH & beta actin controls. Hierarchical organization of the cumulative median CT^−1^ values (inverse CT values were used to depict the expression) identified the ranking of expression as values identified the ranking of expression from high to low as B7-H5, B7-H3, B7-H2, B7-1, PD-L1, B7-2, PD-L2, B7-H6, B7-H4, and B7-H7 (Figure 1A). B7-H5 and B7-H3 showed statistically significant enhanced expression compared to all other B7 family molecules. B7-H2, PD-L1, PD-L2, B7-1, and B7-2 showed comparable expression among themselves while B7-H4, B7-H6, and B7-H7 are expressed at low levels (Figures 1A,B). The immunosuppressive tryptophan degradation pathway (IDO, TDO) is an immunotherapy target to revive anti-tumor immunity (30–32). We set out to identify the mRNA expression levels of IDO and TDO in HCC and our results show that TDO expression is higher than IDO (Figure 1C).

### Dominant Correlative Pattern Among B7 Family Ligands in HCC

**Figure 2:**
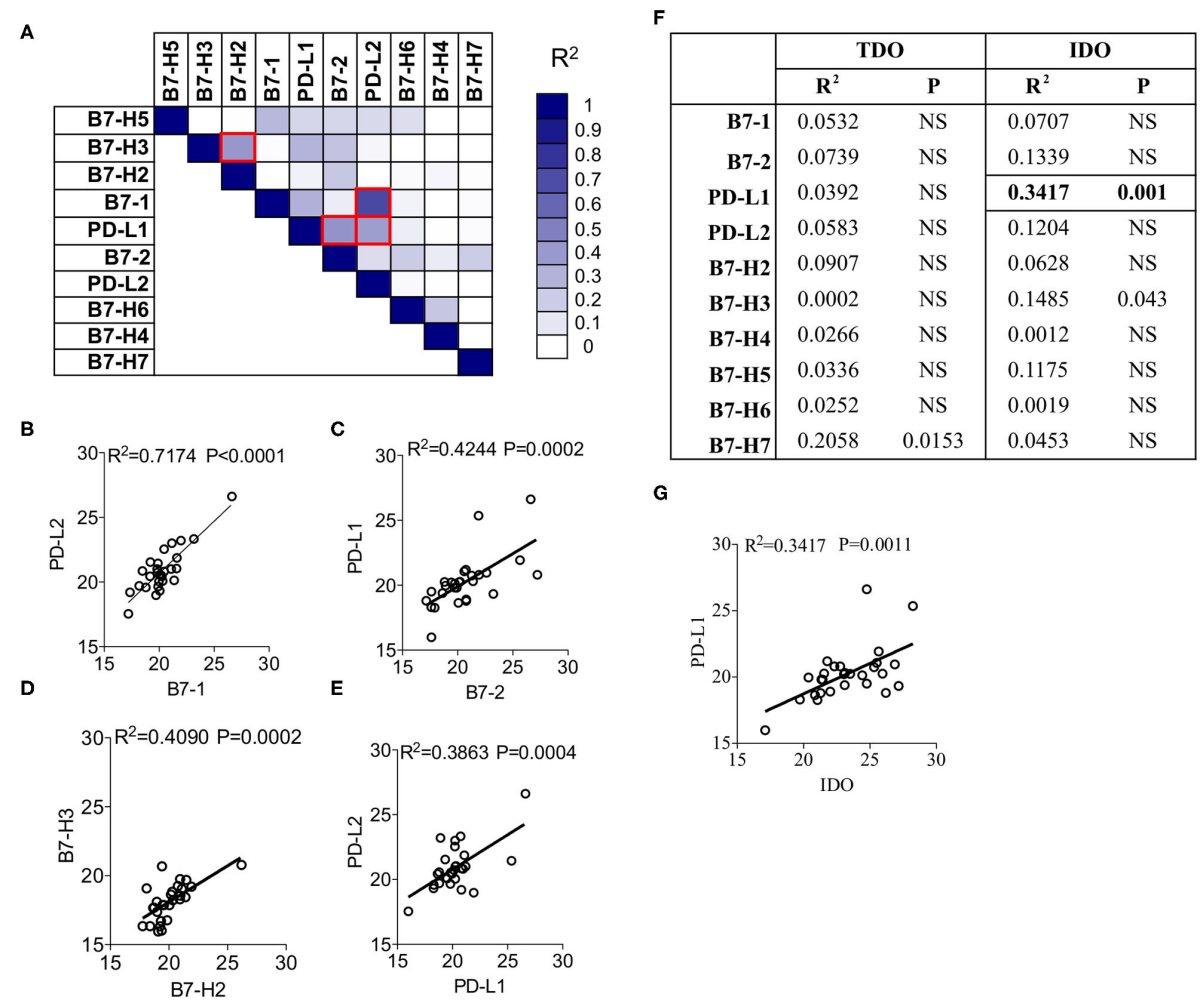
Correlation patterns among B7-Familiy ligands and IDO in the liver of HCC patients. **(A)** CT Values of B7 Family ligands from HCC patients (*n* = 28) were subjected to linear regression analysis among each other. *R*^2^ values were color-coded and red boxes indicate the best correlations based on the hierarchical ranking. Correlative plot with *R*^2^ values above 0.3 are shown for the combination **(B)** B7-1&PD-L2, **(C)** B7-2&PD-L1, **(D)** B7-H2&B7-H3, and **(E)** PD-L2&PD-L1. **(F)** CT values of IDO and TDO were subjected to linear regression analysis with B7 family ligands. Correlative plot with the *R*^2^ values above 0.3 is shown for the combination **(G)** IDO & PD-L1. Linear regression analysis was performed in GraphPad Prism to get *R*^2^ and *P*-values. *R*^2^, Goodness of Fit; P, Significance of the slope deviation from Zero.

**Figure 3:**
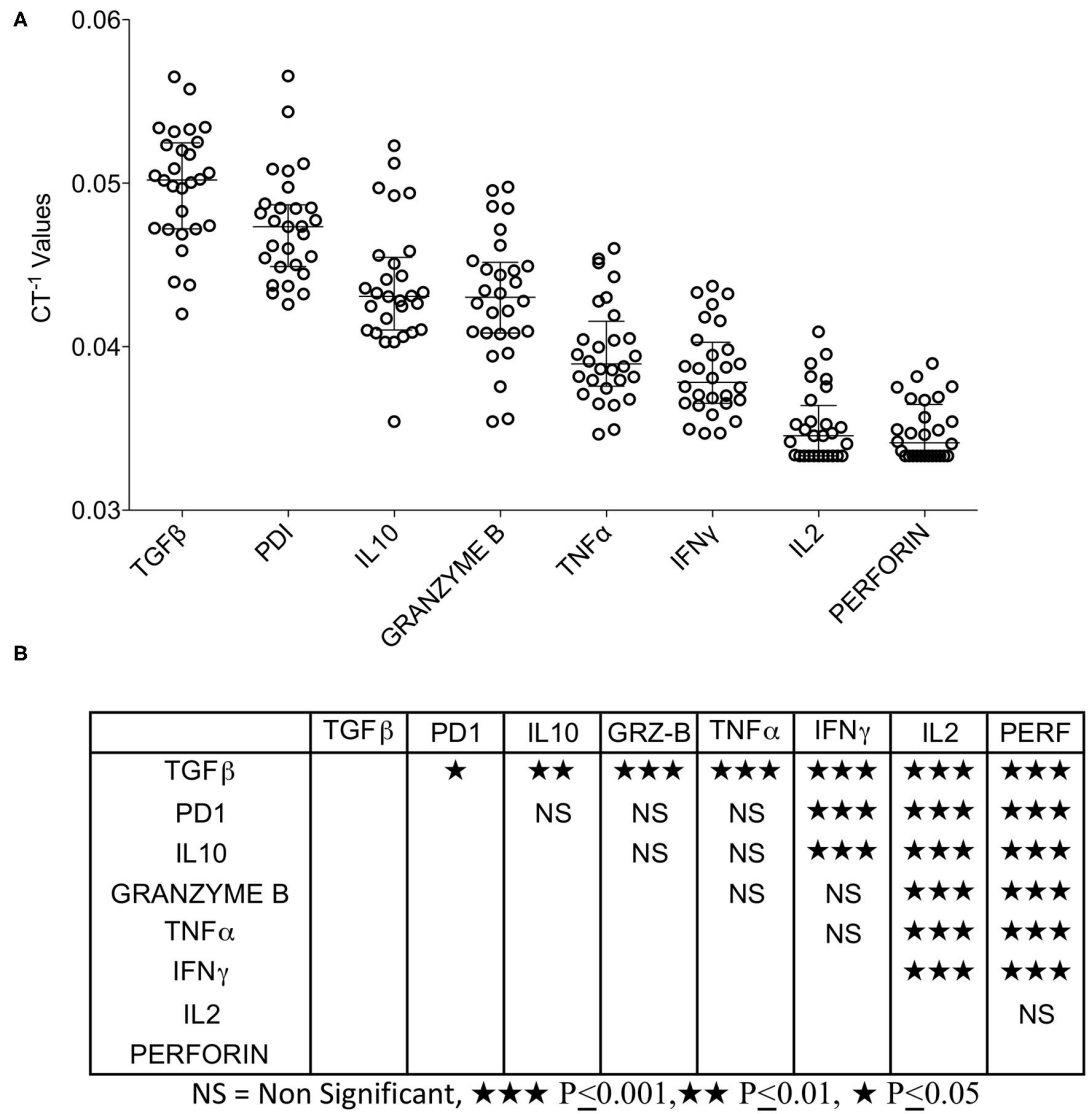
Expression pattern of immune responsive effector molecules in HCC. RNA transcripts of immune responsive effector molecules TGFβ, PD-1, IL-10, IFNγ, TNFα, IL-2, Granzyme B and Perforin were analyzed in Fluidigm^TM^ nanoscale quantitative RT-PCR. **(A)** Inverse CT values (CT^−1^) of immune responsive effector molecules derived from each patient were plotted with the median and range. Median values were organized in hierarchical order of high to low expression. **(B)** One-way ANOVA and Tukey's multiple comparison test was performed to obtain *P*-values. Table shows the statistical significance of the median of CT^−1^ values of each effector molecule in comparison to others. CT values were normalized based on GAPDH & beta actin controls.

To identify the correlation among B7 family ligands, we subjected the CT values of each B7 family ligands in combinations through linear regression analysis. The degree of *R*^2^ correlation values (Example: *R*^2^ = 1 and *R*^2^ = 0 represent the best and no correlation between two molecules, respectively) determines the correlation between two B7 family molecules. Fifty-five combinations tested among the B7 family members and the *R*^2^ values were subjected to hierarchical ranking analysis ranging from best to no correlation. B7-1 expression showed the highest correlation with PD-L2 (*R*^2^ = 0.7174; *P* < 0.0001) (Figures 2A,B). We also identified moderate correlations among three B7 combinations such as (1) B7-2 and PD-L1 (*R*^2^ = 0.4244; *P* = 0.0002) (Figures 2A,C), (2) B7-H2 and B7-H3 (*R*^2^ = 0.4090; *P* = 0.0002) (Figures 2A,D), (3) PD-L1 and PD-L2 (*R*^2^ = 0.3863; *P* = 0.0004) (Figures 2A,E). All other combinations did not show a significant correlation. Altogether, the present analysis identified that B7-1, B7-2, PD-L1, PD-L2, and B7-H3 are correlated in a distinct combinatorial expression pattern.

### Association of IDO and PD-L1 in HCC

To investigate the effect of the expression of IDO and TDO on B7 family ligands, we performed a linear regression analysis between the CT values of IDO, TDO and B7 family ligands. Twenty combinations were investigated to identify *R*^2^ values. Although TDO expression is superior to IDO, it does not correlate with any of the B7 family ligands (Figure 2F). However, IDO shows a trend toward a correlation with PD-L1 (*R*^2^ = 0.3417; *P* = 0.001) (Figures 2F,G). We did not observe any associations between IDO and other B7 family molecules. Altogether, these results demonstrate the correlation between IDO and PD-L1.

### High Expression of TGF-β, IL-10, and PD-1 in HCC

Tumor microenvironment is not only occupied with the co-stimulatory and co-inhibitory ligands but also the immune responsive effector molecules that actually facilitate immune suppression or stimulation. We analyzed the expression of TGF-β, IL-10 (immune suppression), IFNγ, TNFα, IL-2, Perforin, Granzyme B (immune stimulation), and PD-1 (immune exhaustion). We categorized the expression levels of these effector molecules relative to each other by descending hierarchical organization of median CT^−1^ values. Our results demonstrate that TGF-β, PD-1, and IL-10 are expressed at high levels, TNFα and Granzyme B expressed at moderate levels, while IFNγ, IL-2, and Perforin are expressed at very low levels (Figure 3A). Descending hierarchical organization of median CT^−1^ values identified the ranking as TGF-β, PD-1, IL-10, GranzymeB, TNFα, IFNγ, IL-2 and Perforin (Figures 3A,B). Altogether these results demonstrate the dominant expression of immunosuppressive molecules (TGF-β, PD-1, IL-10) in HCC microenvironment.

### Correlation Patterns of Immune Response Genes With B7 Family Ligands

**Figure 4:**
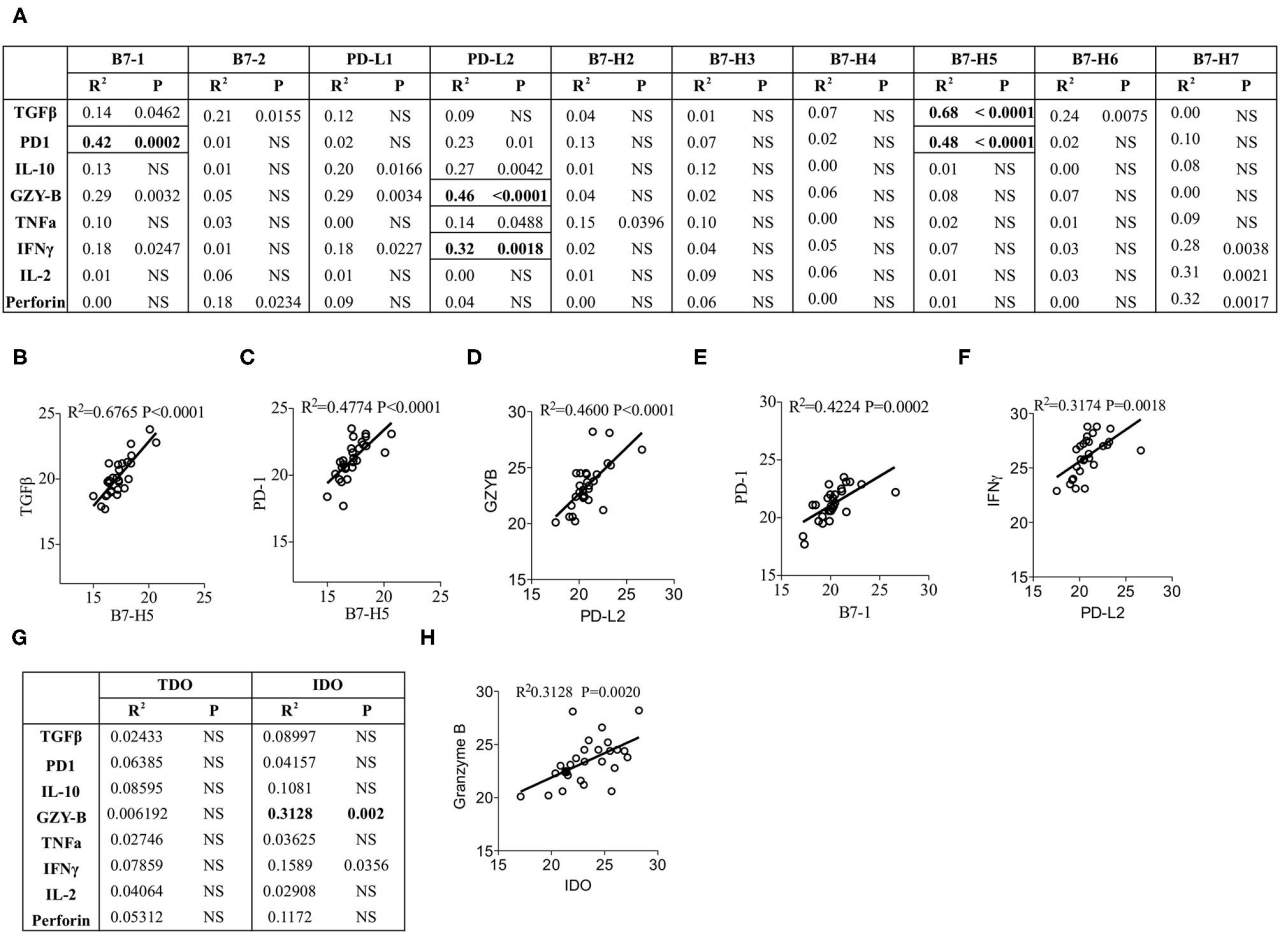
Correlation of B7 family ligands and IDO with immune responsive effector molecules. CT values of each immune responsive effector molecules were subjected to linear regression analysis with the B7 family. **(A)** Correlation B7 family ligands with immune responsive effector molecules is shown in table format. Correlative plots with *R*^2^ values above 0.3 are shown as **(B)** B7-H5&TGFβ, **(C)** PD-1&B7-H5, **(D)** PD-L2&GranzymeB, **(E)** B7-1&PD-1 and **(F)** PDL2&IFNγ. **(G)** IDO and TDO were subjected to linear regression analysis with immune responsive effector molecules to get *R*^2^ and *p*-values. Correlative plots with the *R*^2^ values above 0.3 is shown for the combination **(H)** IDO & Granzyme B. Linear regression analysis was performed in GraphPad Prism to get *R*^2^ and *P*-values. *R*^2^, Goodness of Fit; *P*, Significance of the slope deviation from Zero; NS, Non-significant.

Next we aim to determine the effect of the expression of individual B7 family ligands on the expression of immune responsive effector molecules within HCC microenvironment. CT values of the immune responsive effector molecules were subjected to linear regression analysis with the CT values of B7 family ligands. Eighty combinations were tested to identify *R*^2^ values, which determine the correlation of immune responsive effector molecules with B7 family ligands. Our results identified the best correlation between TGF-β and B7-H5 (*R*^2^ = 0.6765; *P* < 0.0001) (Figures 4A,B). In addition, a statistical trend toward the best correlation was identified with four other combinations. (1) PD-1 and B7-H5 (*R*^2^ = 0.48; *P* < 0.0001), (Figures 4A,C), (2) PD-L2 and Granzyme B (*R*^2^ = 0.46; *P* < 0.0001) (Figures 4A,D), (3) PD-1 and B7-1 (*R*^2^ = 0.4224; *P* = 0.0002) (Figures 4A,E). (4) IFNγ and PD-L2 (*R*^2^ = 0.3174; *P* = 0.0018) (Figures 4A,F).

None of the other combinations of B7 family ligands and immune responsive molecules showed a significant correlation values above 0.3 (Figure 4A). Correlations such as B7-H7 and IL-2, B7-H7 and Perforin were disregarded due to their very low expression levels (Figures 1A, 3A). Altogether, these results demonstrate that although PD-1 is the cognate receptor for the ligands PD-L1 and PD-L2, its expression correlates with other B7 family ligands B7-1 and B7-H5. In addition, the immunosuppressive cytokine TGF-β correlates with B7-H5 while IFNγ and tumorlytic enzyme Granzyme B correlates with PD-L2.

### IDO but Not TDO Correlate With Granzyme B

Next we investigated the correlation of IDO and TDO with immune responsive effector molecules. Linear regression analysis was performed between these two families of molecules with 16 combinations (Figure 4G). Identification of *R*^2^ values demonstrates that TDO expression does not show any correlation with immune responsive effector molecules (Figure 4G). However, IDO expression shows a trend toward correlation with Granzyme B but not with any other immune responsive effector molecules (*R*^2^ = 0.3128; *P* = 0.002) (Figures 4G,H). Altogether these results demonstrate that although TDO is dominant, it does not correlate with immune responsive effector molecules. In contrast, IDO expression correlates with Granzyme B.

### Validation of Correlations Among B7 Family Ligands, Immune Response Genes and IDO After Glypican-3 and Osteopontin Normalization

**Figure 5:**
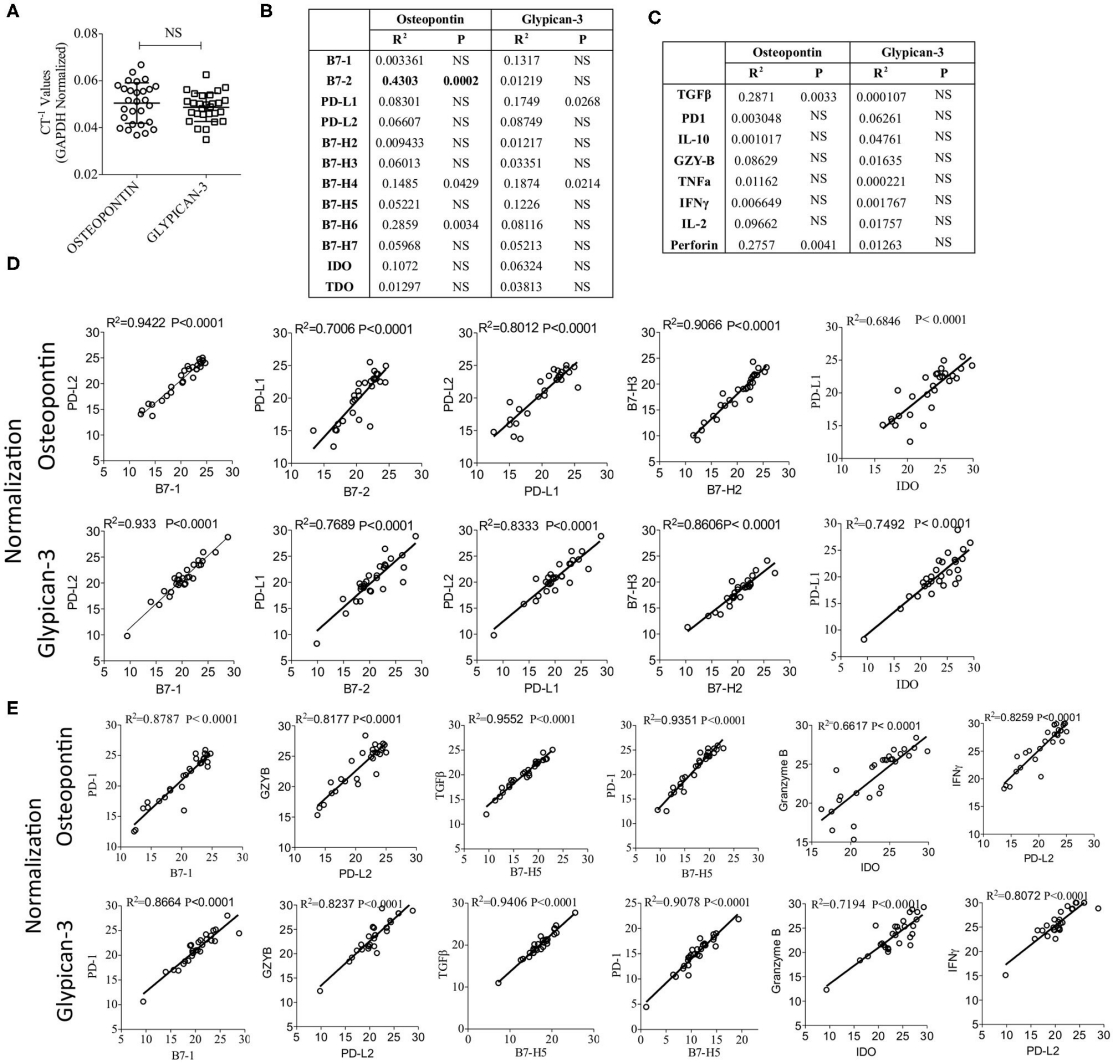
Validation of the identified correlations using HCC biomarkers Osteopontin and Glypican-3. **(A)** Inverse CT values (CT^−1^) of Osteopontin and Glypican-3 derived from each patient, after GAPDH & beta actin normalization, were plotted with median and range. CT values of Osteopontin and Glypican-3 were subjected to linear regression analysis with **(B)** B7 Family ligands, Tryptophan Degrading enzymes and **(C)** immune response genes. Correlation *R*^2^ values and statistical significance are shown in a table format. **(D,E)** GAPDH & beta actin normalized CT values were further normalized based on the expression of Osteopontin or Glypican-3. Correlations that are identified in Figures 2, 4 were further confirmed using Osteopontin and Glypican-3 normalization. **(D)** Correlations between B7 family ligands and IDO. **(E)** Correlations between B7 family ligands, IDO and immune response genes. Linear regression analysis was performed in GraphPad Prism to get *R*^2^ and *P*-values. *R*^2^, Goodness of Fit; *P*, Significance of the slope deviation from Zero; NS, Non-significant.

Glypican-3 and Osteopontin are over expressed in HCC and studies have demonstrated that both of these molecules could serve as the biomarker of HCC progression and could be targeted (37–39). Hence, we determined the mRNA expression levels of Glypican-3 and Osteopontin in HCC samples. Our results show that Glypican-3 and Osteopontin expressions are not significantly different from each other (Figure 5A). In addition, Glypican-3 and Osteopontin did not show significant levels of correlation with B7 family ligands, immune response genes and IDO, except Osteopontin showing a moderate correlation with B7-2 (Figures 5B,C). Next, we normalized CT values based on Osteopontin and Glypican-3 expression and validated the key identified correlation (as identified in Figures 2, 4) that were determined based on GAPDH and beta-actin normalization. We recapitulated and confirmed the identified correlations among B7 family ligands, immune response genes and IDO based on the normalization with HCC associated biomarkers Glypican-3 and Osteopontin (Figures 5D,E).

### IDO Metabolite Kynurenine and PD-L1 Synergistically Inhibit T Cell Responses

**Figure 6:**
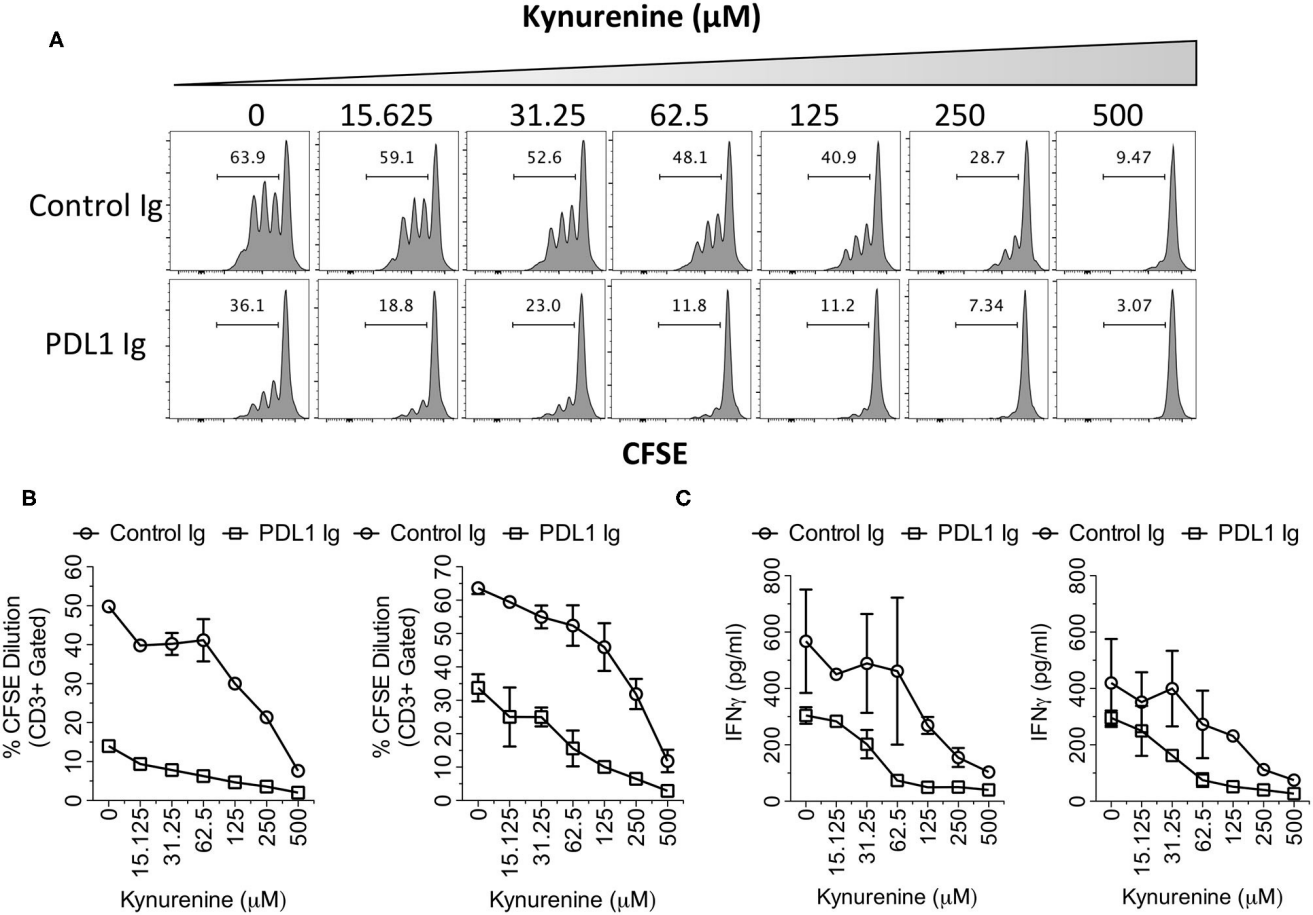
Synergistic inhibitory effects of PD-L1 and Kynurenine on T cell responses. CFSE labeled PBMCs were stimulated on the plates coated with anti-CD3 and anti-CD28 (1 μg/ml) alone or in combination with PD-L1 Ig or Control Ig (10 μg/ml). Subsequently, Kynurenine was added to the culture at a final concentration as indicated. 4 days later, T cell proliferation analysis was performed using flow cytometry. **(A)** Representative CFSE dilution plot is shown. CFSE plots were derived from Forward Scatter, Side Scatter, CD3+ parental gates. **(B)** % CD3+ CFSE dilution is plotted with two independent donors. **(C)** Supernatants of the cultures were analyzed for human IFNγ using ELISA. Concentrations of the IFNγ are shown from two independent experiments using independent donor PBMCs.

IDO correlates only with PD-L1 but not with other B family ligands, which suggests that this correlation is significant. Hence, to investigate the synergistic functional effect of IDO and PD-L1, we tested the effect of PD-L1 Ig/Control Ig and Kynurenine (IDO metabolite) on human peripheral blood mononuclear cells (PBMCs). We stimulated CFSE labeled human PBMCs with plate-bound anti-CD3 & anti-CD28 in the presence of PD-L1 Ig or Control Ig along with the dose escalating concentrations of Kynurenine. Four days later, T cell proliferation was determined by analyzing the percentage of CFSE dilution using flow cytometry. In addition, we also determined the concentrations of secreted Interferon γ (IFNg) in the supernatants of the cultures by Enzyme Linked Immunosorbent Assay (ELISA). Our results demonstrate that PD-L1 Ig inhibits T cell proliferation in the absence of Kynurenine. Similarly, Kynurenine inhibits T cell proliferation in the absence of PD-L1 Ig (Figures 6A,B). In the cultures with both PD-L1 and Kynurenine, the inhibitory effect on T cell proliferation is superior over their individual counterparts (Figures 6A,B). Similarly, both PD-L1 Ig and Kynurenine synergistically inhibit Interferon γ (IFNg) secretion (Figure 6C). Altogether these results demonstrate the synergistic inhibition of PD-L1 and IDO on T cell responses.

## Discussion

Reviving the immune system with immunotherapy is becoming an attractive strategy to treat HCC (40, 41). Immunotherapy agents such as immune checkpoint blocking agents targeting single co-inhibitory molecule show some benefits to patients with HCC (11). These prompted to use second-generation combined immune blocking strategies targeting additional co-inhibitory molecules of the tumor microenvironment. However, practically it is not feasible to test combined immune blocking strategies targeting an array of immunomodulatory molecules with all the combinations in clinical trials or immunotherapy animal model studies. Hence, it is necessary to identify the *in situ* correlation and interaction of immunomodulatory molecules in the tumor microenvironment. We hypothesized that immunomodulatory molecules express in a specific combinatorial pattern in the HCC microenvironment and identification of such *in situ* combinations not only inform the targets for combined immunotherapy approaches but also unveil the immuno physiology of the tumor microenvironment.

Herein, we analyzed the correlative expressions of B7 family molecules and enzymes of tryptophan degradation in the tumor microenvironment of HCC. Consistent with other tumor types (42), we identified the differential expressions of B7 family molecules within the hepatic tumor microenvironment. Using a correlative hierarchical ranking analysis, we identified four best correlations out of 55 combinations tested among B7 family molecules. They are (1) B7-1 and PD-L2, (2) B7-2 and PD-L1 (3) PD-L1 and PD-L2, (4) B7-H2 and B7-H3. Identification of the correlative expression of PD-L1 and PD-L2 suggests that both of these ligands may function together in dampening T cell responses through its receptor PD-1. Clinical trials targeting PD-1 or PD-L1 with monoclonal antibodies showed encouraging results. FDA approved this immunotherapy for the patients who show advanced stage HCC and sorafenib resistance. Although FDA approved this therapy with the contingency that advanced phase clinical trials should demonstrate efficacy, the results are both positive and negative (40, 41). Thus, combined immunotherapy approach may be necessary to demonstrate consistent clinical efficacy. In addition, it has also been demonstrated that the binding structure, kinetics and affinity of PD-L1 and PD-L2 with PD1 are different (43, 44). These structural insights and our observation of the correlation of PD-L1 and PD-L2 suggest that the combined immunotherapy approaches targeting PD-L1, PD-L2 and PD-1 are warranted for advanced HCC management.

We also observed correlations such as (1) B7-1 and PD-L2, (2) B7-2 and PD-L1, (3) PD-1 and B7-1 which are intriguing since PD-L1, PD-L2, PD-1 are co-inhibitory ligands/receptor while B7-1 and B7-2 are dimorphic ligands since they can interact with CD28 or CTLA4 to promote co-stimulation or co-inhibition, respectively. It has been recently demonstrated that B7-1, B7-2 and CD28 co-stimulation is necessary to revive exhausted T cells using anti-PD-1 therapy in animal models and lung cancer patients (45). Thus, both of these two co-stimulatory and co-inhibitory pathways, (1) B7-1, B7-2-CD28 and (2) PD-L1, PD-L2-PD1 may function together in promoting T-cell exhaustion. Our results further support this hypothesis that in the liver tumor microenvironment, B7-1, B7-2, PD-L1, PD-L2 and PD-1 interactions occur extensively, which cumulatively confer dysfunctional anti-tumor immunity. In addition, our data shows the correlation of PD-1 with its non-cognate ligands B7-1 and B7-H5 which further suggests that PD-1 should be targeted in conjunction with other B7 family molecules.

B7-H2 and B7-H3 are co-stimulatory and co-inhibitory molecules, respectively (20, 22). The identification of their correlation suggests that neutralization/dampening of immune activation ensue as a result of the interaction of these molecules. In addition, it is also entirely possible that in the liver tumor microenvironment both of these pathways cumulatively promote T-cell exhaustion/tolerance similar to CD28 and PD-1 (45). Hence, these observations propose that blocking of the inhibitory pathways in HCC microenvironment will not only negate the immunosuppressive signals but also boost the functions of endogenous co-stimulatory molecules, which collectively revive exhausted/tolerized T cells.

B7-H5 plays an important role in the generation of inducible FoxP3+ regulatory T cells through TGF-β (46). Similarly, PD-1 and B7-H5 cumulatively attenuate T cell responses (47). Recent evidences suggest that HCC tumor microenvironment is enriched with Foxp3+CD4+ and PD-1+CD8+ T cells which together provide an extensive immunosuppressive microenvironment (48, 49). In addition, reduction in PD-1 and Foxp3 has been shown to be a predictor of the survival of HCC patients (50). Our observation of the correlation of B7-H5 with TGF-β and PD-1 add to these previous findings that B7-H5 plays an important role in the origin of intrahepatic regulatory T cells and in association with PD1+ T cells, it dampens anti-HCC T cell immunity.

IDO upregulation and its role in immunosuppression have been well established in generic and intrahepatic tumor immunity (51–53). TDO has a predominant homeostatic housekeeping role specific to liver (54), and the observation that TDO overexpression above IDO is due to the indigenous nature of the liver microenvironment. However, none of the B7 family molecules correlate with TDO expression in the liver while IDO shows a correlation with PD-L1 stronger than any other B7 family molecules. Correlation of IDO and PD-L1 has been shown in other tumors (55–57). Preclinical animal model studies also demonstrated that immune checkpoint blocking strategies with IDO targeting show an enhanced anti-tumor responses (58, 59). Our functional investigation on the effect of PD-L1 and IDO metabolite Kynurenine on T cell responses has demonstrated that both Kynurenine and PD-L1 exhibit synergistic inhibitory effect on T cells. Thus, co-blocking of IDO and PD-L1 is a promising strategy to promote anti-HCC T-cell immunity within the liver. However, although several clinical trials are aiming to test combined immune checkpoint blockade agents to revive anti-tumor immunity, some did not show efficacy. For example, a large phase III clinical trial investigating the combined checkpoint blockade of IDO and PD-L1 did not show efficacy in melanoma patients (60). This suggests that additional immune suppression persists beyond IDO and PD-L1 which neutralizes anti-tumor immunity. We have identified additional immunomodulatory pathways that coexist beyond IDO and PD-L1 suggesting that HCC tumor microenvironment is complex. Further mechanistic and functional studies are warranted to identify which pathways are dominant and superior over others in reviving anti-HCC immunity.

Granzyme B is a tumorlytic effector enzyme, which is predominantly used by cytotoxic lymphocytes and natural killer cells to lyse tumor cells (61, 62). In addition, Granzyme B+ lymphocytes were shown to be significantly associated with improved survival of HCC patients (63). We observed a correlation of Granzyme B with PD-L2 and IDO. IDO plays an important role in conferring adaptive tumor resistance to immune lysis since animal model studies have demonstrated that sole blocking of PD-1 or CTLA4 pathway enhances the IDO mediated tumor resistance. However, co-blocking of IDO and PD-1/CTLA4 significantly abolish tumor progression (58, 59). Similarly HCC patients treated with single immune checkpoint blocking antibodies to PD-1 or PDL-1 are always not responsive to therapy (40, 41). Thus, we speculate that although Granzyme B is tumorlytic, both PD-L2 and IDO may override and neutralize Granzyme B+ lymphocyte activity. This also proposes that both PDL1 and IDO may need to be targeted to enhance Granzyme B tumorlytic activity. Altogether, our data suggest that co-blocking of IDO and B7 family is a promising strategy to promote anti-HCC T-cell immunity without adaptive tumor resistance.

A limitation of our present study is the investigation of the correlation of B7 family molecules, Tryptophan degrading enzymes and immune effectors only at RNA but not protein expression levels. Although significance of our approach is the identification of correlation pattern by combination analysis, such an approach at protein levels is laborious and often limited with the sensitivity to demonstrate correlation. Nevertheless, our results were supported by previous studies which demonstrated that some B7 family molecules such as PD-L1 (64, 65), PD-L2 (66), PD-1 (67), B7-H2 (68), B7-H3 (69) and IDO (70) play a significant functional role in modulating anti-HCC immunity. Although we have demonstrated the synergistic functions of IDO and PD-L1, future studies are necessary to determine if the other identified correlation of costimulatory and coinhibitory molecules play a functional synergistic role in modulating anti-HCC immunity.

Liver is a tolerogenic organ rich in parenchymal and non-parenchymal cell types such as Hepatocytes, Kupffer Cells, Liver Sinusoidal Endothelial Cells, Hepatic Dendritic Cells, Hepatic Stellate Cells, Mesenchymal Stromal Cells and Hepatic B cells that constantly interact with T cells in order to execute immune tolerance. Upon injury, the resting status of these intrahepatic antigen presenting cells gets compromised leading to inflammation followed by fibrosis/cirrhosis. Eventually, immune suppression prevails that leads to HCC and significance of these cell types in HCC progression is important. Our earlier study has demonstrated that Hepatic Stellate Cells upregulate PD-L1 and IDO by IFNγ while blocking of IDO activity completely abolishes their immunosuppressive potential (71). Similarly, Kupffer cells in HCC were shown to execute immune suppression through PD-L1-PD-1 pathways (72). In the present study, we observed a correlation between PD-L2 and IFNγ. Future studies are warranted to define the coexpression, regulation and functions of B7 family molecules and Tryptophan degrading enzymes in intrahepatic APCs derived from HCC microenvironment. In conclusion, our study provided evidence that co-stimulatory and co-inhibitory molecules exhibit specific correlation pattern among themselves, with IDO and immune responsive effector molecules. This information is important to inform second-generation immunotherapy approaches for liver cancer management.

## Data Availability Statement

All datasets generated for this study are included in the article/supplementary material.

## Ethics Statement

The studies involving human participants were reviewed and approved by Emory University IRB. The ethics committee waived the requirement of written informed consent for participation.

## Author Contributions

RC conceived, designed the research plan, performed most experiments, analyzed results, and wrote the manuscript. RS performed experiments related to flow cytometer and ELISA. OA, DR, AF, and BE provided patient materials. DA and GG helped with qPCR array. All authors contributed to the article and approved the submitted version.

## Conflict of Interest

The authors declare that the research was conducted in the absence of any commercial or financial relationships that could be construed as a potential conflict of interest.
